# Outcomes of phosphorus-32 microparticle intratumoral implantation added to chemotherapy in patients with metastatic pancreatic adenocarcinoma

**DOI:** 10.1016/j.igie.2024.06.005

**Published:** 2024-07-02

**Authors:** Amanda Huoy Wen Lim, Nimit Singhal, Dylan Bartholomeusz, Joshua Zobel, Jeevinesh Naidu, William Hsieh, Benjamin Crouch, Harpreet Wasan, Daniel Croagh, Adnan Nagrial, Morteza Aghmesheh, Edmund Tse, Christopher K. Rayner, Nam Quoc Nguyen

**Affiliations:** 1 Gastroenterology and Hepatology, Royal Adelaide Hospital, Adelaide, South Australia, Australia; 2 The University of Adelaide, Adelaide, South Australia, Australia; 3 Oncology, Royal Adelaide Hospital, Adelaide, South Australia, Australia; 4 Department of Nuclear Medicine, Royal Adelaide Hospital, Adelaide, South Australia, Australia; 5 Oncology, Imperial College Healthcare NHS Trust, London, United Kingdom; 6 Upper Gastrointestinal and Hepatobiliary Surgery, Monash Medical Center, Clayton, Victoria, Australia; 7 Oncology, Westmead Hospital, Westmead, New South Wales, Australia; 8 Oncology, Wollongong Hospital, Wollongong, New South Wales, Australia

## Abstract

**Background and Aims:**

Metastatic pancreatic ductal adenocarcinoma (mPDAC) has a 5-year survival rate of 3%. In nonmetastatic, locally advanced pancreatic cancer, the addition to chemotherapy of EUS-guided intratumoral phosphorus-32 (^32^P) microparticle implantation has achieved good local disease control. The aim of this study was to report the clinical outcomes of this treatment in patients with mPDAC.

**Methods:**

Patients with mPDAC treated with chemotherapy and intratumoral ^32^P-microparticles from 5 centers in Australia and the United Kingdom were analyzed retrospectively.

**Results:**

Fourteen patients were treated (7 female subjects; median age, 64.5 years; Eastern Cooperative Oncology Group performance status scores 0/1/2, 21.4%/57.1%/21.4%). The median baseline primary tumor longest diameter was 40.5 mm. Patients had a median of 3 metastases (interquartile range, 2.25 to 5) and received either 5-fluorouracil, leucovorin, irinotecan, and oxaliplatin (n = 4) or gemcitabine/nab-paclitaxel (n = 10). ^32^P microparticles were implanted at a median 3.1 months from chemotherapy commencement. Local disease control rate at 3 months’ postimplantation was 100%. Primary tumor longest diameter decreased by 25% (interquartile range, –31.4% to –16.7%; *P* = .008), and serum cancer antigen 19-9 levels declined from 134 U/mL to 66 U/mL (*P* = .018). Local progression-free survival was 12.2 months (95% CI, 9.0-15.4 months) from chemotherapy commencement and 8.3 months (95% CI, 2.6-16.0 months) from ^32^P microparticle implantation. Therapy was associated with improved quality of life, including global health status at 12 weeks’ postimplantation (*P* = .037). Median overall survival was 13.8 months (95% CI, 10.5-17.1 months) from chemotherapy commencement and 11 months (95% CI, 5.6-17.4 months) from ^32^P microparticle implantation. No grade 4/5 acute toxicities were observed.

**Conclusions:**

This first multicenter analysis of combined chemotherapy and EUS-guided ^32^P microparticle implantation in mPDAC shows the potential clinical benefits of local tumor control in a cohort for whom outcomes are historically poor.

Pancreatic cancer is projected to become the second leading cause of cancer death before 2030^1^^,^^2^; its aggressive nature and poor survival rate have resulted in a significant global burden of disease. Pancreatic ductal adenocarcinoma (PDAC) accounts for 80% of pancreatic cancer and is often diagnosed at a late stage due to early metastasis, lack of markers for early detection, and an absence of proven screening programs. More than one-half of patients have metastatic pancreatic ductal adenocarcinoma (mPDAC) at presentation, which carries a dire 5-year survival rate of 3%.^3^

Standard therapy for mPDAC is chemotherapy consisting of 5-fluorouracil, leucovorin, irinotecan, and oxaliplatin (FOLFIRINOX), gemcitabine alone, or gemcitabine and nab-paclitaxel (Gem/NabP). For many years, gemcitabine was the mainstay of treatment, but phase 3 studies have shown survival benefits with FOLFIRINOX and Gem/NabP compared with gemcitabine alone (FOLFIRINOX median overall survival, 11.1 vs 6.8 months [*P* < .001]; Gem/NabP median overall survival, 8.5 months vs 6.7 months [*P* < .001]).^4^^,^^5^ However, conventional chemotherapy may not be sufficient for local disease control.

Locally advanced disease has been shown to be the cause of 30% of deaths in patients with pancreatic cancer, indicating the importance of locoregional control.^6^ One of the reasons systemic chemotherapy alone may not be effective for controlling local disease is that, unlike other solid tumors, pancreatic cancer harbors a hypoxic microenvironment.^7^^,^^8^ Indeed, pancreatic cancer hypoxia represents an independent prognostic factor, and when hypoxic areas are identified (using an Eppendorf electrode) within the tumor, the prognosis is worse than for well-oxygenated tumors.^8^^,^^9^ Some studies have shown that radiotherapy can restore tumor vasculature, thereby reducing intratumoral hypoxia and potentiating the effect of chemotherapy.^10^, ^11^, ^12^, ^13^, ^14^ This may explain why, in addition to cancer cell injury, radiotherapy in combination with chemotherapy can improve local tumor control.^15^

Radiotherapeutic options in PDAC include conventional radiotherapy (most commonly external beam or intensity-modulated radiation therapy) and stereotactic body radiation therapy (SBRT).^16^ In mPDAC, SBRT to the primary tumor has shown good local control rates.^17^^,^^18^ Although this has not yet been shown to translate to extended overall survival, an improvement in pain with no deterioration in quality of life (QoL) has been reported.^19^ Patients with pancreatic cancer often have poor QoL due to pain, jaundice, and weight loss, as well as the need for multiple hospital visits.^19^^,^^20^ With limited life expectancy in mPDAC, improving symptoms and QoL is an important goal.

A novel type of radiation therapy is brachytherapy, whereby radioisotopes are placed inside or near the tumor. Brachytherapy has been used in prostate, breast, and particularly gynecologic cancers.^21^, ^22^, ^23^ ^125^Iodine seeds have been placed via interstitial needles with good results (clinical control up to 87%) in patients with low-risk and selected intermediate-risk prostate cancer.^24^, ^25^, ^26^ In patients with unresectable pancreatic cancer, implantation of ^125^iodine seeds has been shown to be feasible and safe, and to achieve pain relief, although an effect on survival has not been shown.^27^

Another radioisotope used for brachytherapy in PDAC is phosphorus-32 (^32^P) delivered as ^32^P microparticles (OncoSil; OncoSil Medical Ltd, North Sydney, NSW, Australia) comprising an alloy of silicon and phosphorus activated in a nuclear reactor and suspended in a diluent. The ^32^P microparticles are administered by injection through an EUS-guided needle. ^32^P emits beta radiation, which travels only a short distance (<1 cm) within tissue and has a half-life of 14.3 days.^28^ In pancreatic carcinoma xenografts in nude mice, intratumoral ^32^P treatment resulted in complete response in tumors in both 1.0 and 2.0 MBq treatment groups.^29^ When combined with chemotherapy for nonmetastatic locally advanced PDAC, it has shown promising results, with 1 study reporting a 42% downstaging to surgical resection, whereas a 50-patient pilot study found a 90.5% local disease control rate (LDCR) at 16 weeks’ postchemotherapy commencement.^30^^,^^31^ To our knowledge, the utility of ^32^P microparticle implantation in patients with mPDAC has never been investigated. The current article reports the first multicenter analysis of this form of brachytherapy in combination with systemic chemotherapy in patients with mPDAC.

## Methods

We undertook a multicenter retrospective analysis of prospectively gathered data of all patients with mPDAC who received combination chemotherapy and EUS-guided intratumoral ^32^P microparticles (OncoSil) from September 2017 to September 2020. Patients were treated in 5 centers in Australia and the United Kingdom, and they received ^32^P under either the PanCO^31^ study protocol (but had metastases identified post-enrollment by use of positron emission tomography imaging) or via a special access scheme. The implanted device comprises OncoSil ^32^P microparticles and OncoSil Diluent. In therapeutic use, 98% of the total radiation is delivered within 81 days. The implanted device is supplied sterile and is intended for single patient use. Before study participant administration, the implanted device is prepared by the treating facility’s radiopharmacist. ^32^P activity was calculated by using the patient’s target tumor volume to deliver a 100 Gy absorbed dose (calculated before the procedure to achieve an implanted volume/tumor volume ratio of 8%).

The delineation and prediction of gross tumor volume on medical images were used to form the primary tumor target volume. The dose to be implanted (in megabecquerels) is calculated as implanted volume (in milliliters) × 6.6. Each individual vial is placed inside a Perspex-lined lead pot to shield personnel from the radiation during handling and shipping. Before suspension preparation, the microparticles and diluent are stored at a temperature between 15°C and 25°C. Once prepared, the implantable device is stored between 15°C and 25°C and within a Perspex-lined lead pot; it is used within 24 hours of suspension preparation.

**Figure 1:**
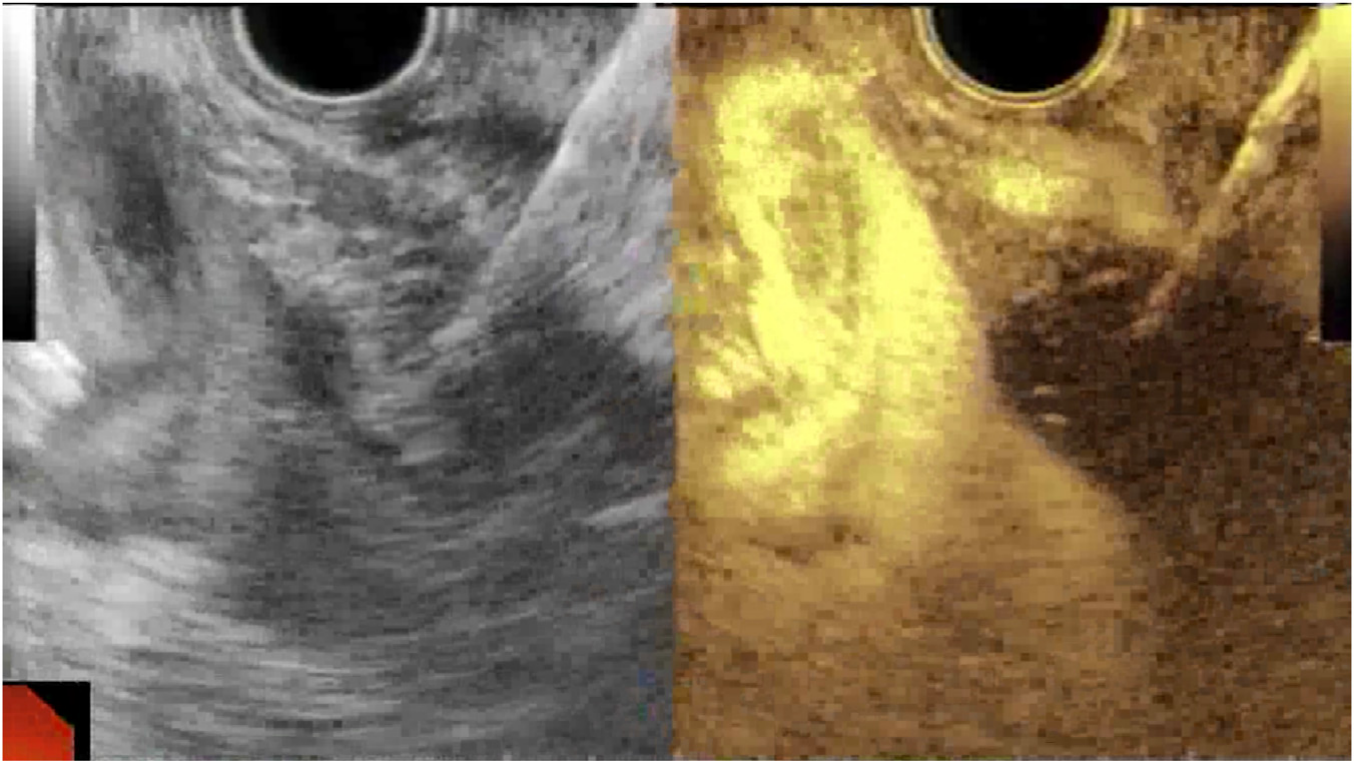
Insertion of a 22-gauge FNA needle into the center of the tumor for phosphorus-32 implantation using EUS and contrast-enhanced harmonic EUS guidance.

Via EUS, using color Doppler to avoid any intervening vessels, a 22-gauge EchoTip FNA needle (Cook Medical, Bloomington, Ind, USA) is inserted into the pancreatic tumor (Fig. 1). If the lesion is <2 cm, the total implanted volume is inserted into the center of the lesion. If the lesion is ≥2 cm, 50% will be inserted into the center, and 25% of the volume will be inserted on either side. This is followed by a 1 mL saline flush. After injection of the material, the needle sheath is pushed out into the gastric lumen and the needle retracted into the sheath., Saline is subsequently used to flush the sheath into the gastric lumen. The sheath, scope, and needle complex are then removed from the patient, and further flushing is performed into a biohazard disposal container. After ^32^P microparticle implantation, bremsstrahlung whole-body planar plus single-bed single-photon emission CT imaging was performed within 4 hours to confirm intratumoral distribution of the microparticles.

The study protocol was approved by the Central Adelaide Local Health Network Human Research Ethics Committee (HREC 16785).

### Patient selection

Patients were eligible if they were ≥18 years of age, had histologically confirmed PDAC with evidence of metastases on imaging before ^32^P microparticle implantation, and received chemotherapy. Patients who had undergone previous radiotherapy for PDAC were excluded.

### Data collection

Baseline patient demographic characteristics were recorded, including the Eastern Cooperative Oncology Group (ECOG) performance status score and the Charlson Comorbidity Index score. Tumor size, total size of metastases, and tumor markers (cancer antigen 19-9 [CA 19-9]) were documented at baseline and serially thereafter. Oligometastatic disease was defined according to the criteria of Damanakis et al.^32^ Implantation success was defined as successful injection of ^32^P microparticles visualized via EUS and evidence of intratumoral distribution on the bremsstrahlung scan. Clinical response was defined according to the Response Evaluation Criteria in Solid Tumors (RECIST) 1.1 criteria.^33^ LDCR was defined as stable disease, partial response (PR; 30% decrease in sum of longest diameters [LDs] of target lesions), or complete response (disappearance of lesions). Progressive disease was defined as a ≥20% increase in the sum of LD of the target lesions or the presence of new lesions.

Local disease adverse events were recorded, including biliary obstruction and gastric outlet obstruction. Progression-free survival (PFS) was calculated as the time interval from chemotherapy commencement and from ^32^P implantation to death or disease progression after ^32^P implantation. Overall survival was calculated from chemotherapy (regimen given with ^32^P implantation) commencement and from ^32^P implantation to death.

Quality of life (QoL) was assessed on the day of or before ^32^P implantation and at 12 weeks’ postimplantation using the European Organisation for Research and Treatment of Cancer QLQ-C30 questionnaire. This questionnaire is used to assess a 2-item global health status/QoL scale, 5 functional scales (physical, role, cognitive, emotional, and social functioning), 3 symptom scales (fatigue, pain, and nausea/vomiting), and 6 single-item scales (dyspnea, insomnia, appetite loss, constipation, diarrhea, and financial difficulties).^34^ All scales were scored from 0 to 100; high scores for global health status/QoL and functional scales reflect better functioning and QoL, but high symptom scale scores reflect increasing severity of symptoms.

### Statistical analysis

The primary outcome was change in primary tumor size. Secondary outcomes included clinical response at 3, 6, 9, and 12 months after ^32^P implantation; change in CA 19-9; overall survival and PFS; adverse event (AE) rate; and adverse events from local disease progression. Outcomes were reported as means ± standard deviation for normally distributed variables or medians (interquartile range [IQR]) for variables that were not normally distributed. Paired variables that were normally distributed were analyzed by using paired Student t tests; variables that were not normally distributed were analyzed by using the Wilcoxon signed rank test. PFS and overall survival were assessed with Kaplan-Meier survival analyses.

Analyses were performed by using IBM SPSS version 27 (IBM SPSS Statistics, IBM Corporation, Armonk, NY, USA). *P* < .05 (2-tailed) was considered statistically significant.

## Results

Fourteen patients with mPDAC (7 female subjects; median age, 64.5 years [IQR, 58-68 years]) were included in the analysis (Table 1). The majority of patients had an ECOG performance status score of 1 (57.1%), although 3 patients had an ECOG performance status score of 2 (21.4%). Primary tumors were largely located in the pancreatic head (50%) with a median LD of 44 mm (IQR, 32.5-58.5 mm). Oligometastatic disease was present in 28.6% of patients. Patients had a median of 3 metastases (IQR, 2.25-5), and the median total sum of LD for metastases was 27.5 mm (IQR, 16-46.5 mm).

**Table 1:** Baseline demographic and tumor characteristics (N = 14)

Characteristic	Results
Female sex	7 (50)
Age, y	64.5 (58-68)
Race	
White	9 (64.3)
Mediterranean	2 (14.3)
Asian	2 (14.3)
Black	1 (7.1)
ECOG performance status score∗	
0	3 (21.4)
1	8 (57.1)
2	3 (21.4)
Charlson Comorbidity Index score	8 (8-8.75)
Primary tumor stage	
T1	0 (0)
T2	2 (14.3)
T3	7 (50)
T4	5 (35.7)
Nodal status	
N0	7 (50)
N1	7 (50)
Oligometastatic	4 (28.6)
Pancreatic tumor location	
Head	7 (50)
Neck	1 (7.1)
Body	5 (35.7)
Tail	1 (7.1)
Primary tumor, longest diameter, mm	44 (32.5-58.5)
Tumor volume, cm^3^	21.8 (14.3-34.4)
Site(s) of metastatic disease	
Liver	6 (42.9)
Lung	5 (35.7)
Liver + lung	1 (7.1)
Liver + peritoneal	1 (7.1)
Lung + peritoneal	1 (7.1)
No. of metastases	3 (2.25-5)
Largest metastasis, mm	14.3 (9-20.5)
CA 19.9, U/mL	
In all patients (n = 13)	134 (66-3380)
In patients with baseline >35 U/mL [n=10]	857 (103-3456)

Values are n (%) or median (interquartile range).

*ECOG*, Eastern Cooperative Oncology Group; *CA 19-9*, cancer antigen 19-9.

∗ ECOG performance status scores range from 0 to 5, with higher scores indicating greater disability.

### Chemotherapy and intratumoral ^32^P microparticle implantation

Patients underwent ^32^P microparticle implantation in combination with FOLFIRINOX (n = 4; 1 as second line) or Gem/NabP (n = 10; 2 as second line) chemotherapy. A median 1.6 mL (IQR, 1.1-2.38 mL) of ^32^P (median activity, 11.63 MBq; IQR, 7.54-17.85 MBq) was injected at a median of 3.1 months (IQR, 1.1-4.9 months) from the initial commencement of chemotherapy. There was a 100% implantation success rate. There were no AEs, either immediate or within 7 days of ^32^P implantation.

### Response before implantation

Nine patients underwent ^32^P microparticle implantation >2 months after commencement of chemotherapy. There was a median –7.4% (IQR, –0.13% to 0%) maximal change in primary tumor LD from baseline to preimplantation. Two patients (14.3%) had a PR at preimplantation (per RECIST criteria), and 3 (22.2%) patients had progressive disease.

### Primary end point: change in tumor size

**Figure 2:**
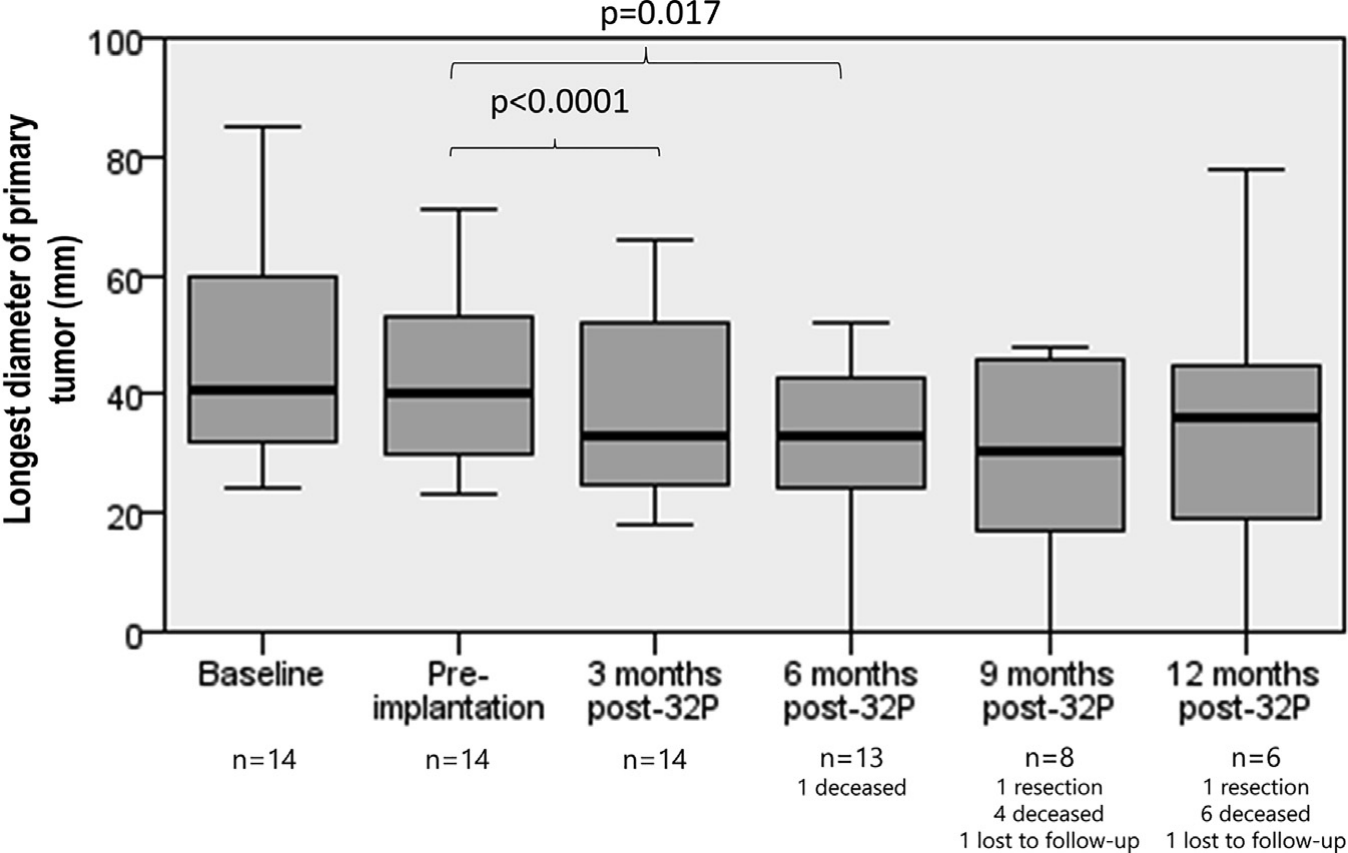
Box plot of primary tumor longest diameter over time. *32P*, Phosphorus-32.

Overall, there was a median maximal reduction in primary tumor LD from preimplantation of 25% (IQR, –31.4% to –16.7%; *P* = .008), with significant reductions in primary tumor LD at 3 and 6 months’ postimplantation (Fig. 2).

### Secondary end points: response, local disease control, and survival

Over a median follow-up period of 13.9 months (IQR, 10.5-20.3 months) from the date of chemotherapy commencement, the best RECIST local tumor response was 8 (57.1%) PR and 6 (42.9%) stable disease. CA 19-9 levels dropped from a median of 134 U/mL preimplantation to 66 U/mL at nadir (*P* = .018).

LDCR at 3 months after ^32^P implantation was 100% (3 PR and 11 stable disease). This includes one PR in a patient who displayed local disease progression before implantation. LDCR at 6, 9, and 12 months was 78.6% (n = 11 of 14; 1 deceased), 50% (n = 7 of 14; 6 deceased), and 50% (n = 7 of 14; 6 deceased), respectively.

**Figure 3:**
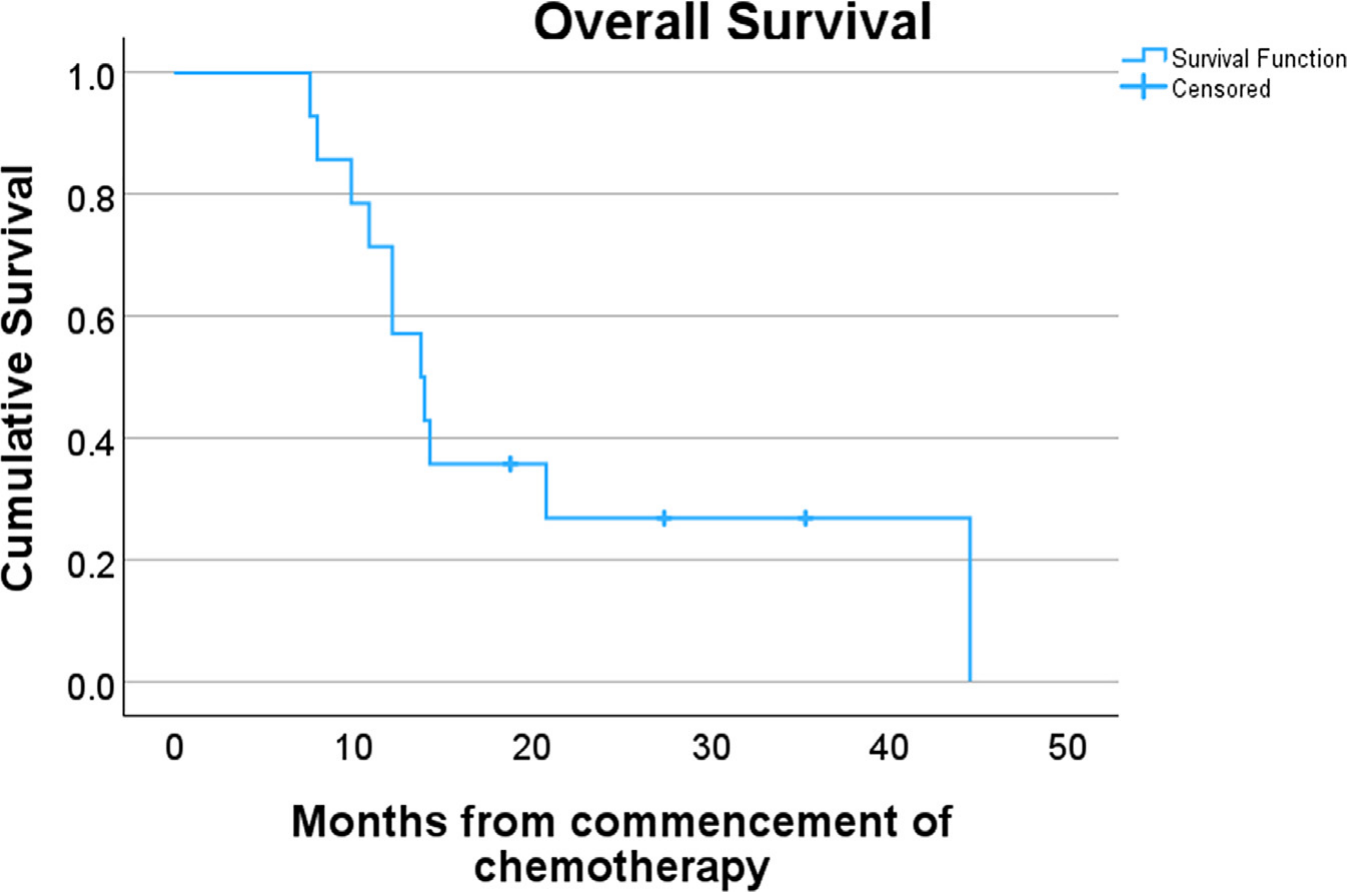
Kaplan-Meier curve showing overall survival for all patients (N = 14) from commencement of chemotherapy.

Median PFS from chemotherapy commencement was 9.2 months (95% CI, 5.7-12.7 months); from ^32^P implantation, it was 5.9 months (95% CI, 4.6-7.2 months). Local PFS was 12.2 months (95% CI, 9-15.4 months) from chemotherapy commencement and 8.3 months (95% CI, 2.6-16.04) from ^32^P implantation. The site of first progression was distant in 57.1% (n = 8), local and distant in 28.6% of patients (n = 4), and local in 7.1% (n = 1); 7.1% (n = 1) had no progression. One patient (7.1%) had a remarkable response in both local and distant disease, leading to disease downstaging with undetectable liver metastases on follow-up imaging. Thus, the patient underwent surgical resection 6.3 months’ post-chemotherapy and achieved R0 resection. This was the only patient with first local progression, with recurrence in the surgical bed at 22 months’ post-chemotherapy commencement. Only 1 patient (7.1%) had local disease progression that resulted in biliary and gastric outlet obstruction requiring insertion of biliary and duodenal stents 42.5 months’ post-chemotherapy commencement; 7 patients (50%) had pre-existing biliary stents. The median survival times for all patients from chemotherapy commencement and ^32^P implantation were 13.8 months (95% CI, 10.5-17.1 months) and from ^32^P implantation was 11.5 months (95% CI, 5.6-17.4), respectively (Fig. 3).

### Adverse events

No grade 4/5 AEs were observed. Four patients (28.6%) had 6 chemotherapy-related grade 3 AEs (neuropathy, neutropenia, anemia, infection), which were not related to the ^32^P microparticle device or the implantation procedure. Importantly, 4 of these AEs occurred before implantation, whereas 1 occurred 3 months’ postimplantation (neuropathy), and 1 occurred >6 months’ postimplantation (infection). Three patients (21.4%) had grade 1/2 AEs related to either chemotherapy, the ^32^P microparticles, or the implantation procedure (abdominal pain, nausea, fatigue, and dyspepsia). These AEs occurred 2 weeks’ to 2 months’ postimplantation.

### Quality of life

Table 2 depicts the European Organisation for Research and Treatment of Cancer QLQ-C30 scores on the day of or day before implantation and 12 weeks’ postimplantation. Clinically relevant improvements (ie, 10-point difference or higher) were noted in global health status, physical, role, emotional and cognitive functioning, fatigue, pain, and insomnia. This was positive and statistically significant for global health status/QoL (*P* = .037). There was no statistically significant difference in pain score (*P* = .373).

**Table 2:** EORTC QLQ-C30 scale scores at preimplantation and 12 weeks’ postimplantation

EORTC QLQ-C30 scale	Median score (/100) preimplantation	Median score (/100) 12 weeks’ postimplantation	*P* value
Global health status/QoL	50.0	66.7	.037
Physical functioning	66.7	80.0	.108
Role functioning	66.7	83.3	.893
Emotional functioning	66.7	77.7	.292
Cognitive functioning	83.3	100	.257
Social functioning	66.7	66.7	.887
Fatigue	44.4	33.3	.200
Nausea and vomiting	16.7	16.7	.496
Pain	33.3	16.7	.373
Dyspnea	33.3	33.3	.655
Insomnia	66.7	33.3	.059
Appetite loss	33.3	33.3	.066
Constipation	0.00	0.00	1.000
Diarrhea	0.00	0.00	.527
Financial difficulties	0.00	33.3	.414

*EORTC*, European Organisation for Research and Treatment of Cancer; *QoL*, quality of life.

## Discussion

With a median overall survival of <1 year, the pursuit for new and innovative treatment options for mPDAC remains a priority.^5^^,^^35^, ^36^, ^37^, ^38^ Our study of a cohort of patients with mPDAC treated with EUS-guided ^32^P microparticles has shown a decrease in primary tumor size until 9 months after ^32^P implantation with a 100% 3-month LDCR. This finding, in addition to a local PFS of >1 year, supports the benefits of adding ^32^P microparticles to standard-of-care chemotherapy for local disease control and prevention of death or AEs due to local disease progression.

Furthermore, a higher median overall survival from ^32^P implantation has been shown compared with overall survival historically reported in mPDAC. This is despite overall survival calculated from ^32^P implantation that usually occurs after several cycles of chemotherapy. Although PFS was 5.9 months, progression was largely distant, with an encouraging local PFS from implantation of 8.3 months. Two pivotal trials of systemic chemotherapy regimens for mPDAC showed a median overall survival of up to 11.1 months from the date of randomization,^4^^,^^5^ with a median PFS of 6.4 months (95% CI, 5.5-7.2 months) in patients treated with FOLFIRINOX and 5.5 months (95% CI, 4.5-5.9 months) in those treated with Gem/NabP.^4^^,^^5^ Other studies have shown similarly poor outcomes in patients with mPDAC.^35^^,^^39^

In addition to local tumor control, radiation can induce local and systemic immunotherapeutic effects, which may have played a role in the encouraging local control and overall survival observed in the current study.^40^^,^^41^ A possible explanation for the success of our approach, which is in contrast to the lack of improvement in survival with conventional radiotherapy and SBRT, is that ^32^P microparticle implantation provides a higher radiation dose (100 Gy). Conventional radiotherapy delivers 40 to 60 Gy to the tumor, with the dose limited by the tolerability of large-field irradiation of the stomach and duodenum.^42^^,^^43^ Although SBRT enables more precise delivery of high doses of radiation to small tumor volumes and therefore results in lower toxicity compared with conventional radiotherapy, the overall dose is similar, involving 25 to 45 Gy over 5 fractions.^43^, ^44^, ^45^, ^46^

In addition, chemotherapy given during conventional radiotherapy or SBRT typically comprises single-agent regimens (eg, gemcitabine, 5-fluorouracil, capecitabine), potentially compromising systemic control of metastatic disease, rather than a multi-agent approach (FOLFIRINOX; Gem/NabP) used as standard-of-care in mPDAC. SBRT also requires prior fiducial placement and planning using CT imaging, which can delay treatment.^16^^,^^47^, ^48^, ^49^ However, ^32^P microparticles in suspension can be administered in conjunction with ongoing standard-of-care multiagent chemotherapy without adversely affecting systemic control.

Although no change in pain score was seen, there was an improvement in QoL at 12 weeks’ postimplantation compared with immediately before implantation, which might be related to locoregional tumor control. Despite the latter being better with SBRT, QoL has been shown to remain static post-SBRT in pancreatic cancer, potentially attributable to the negative impact of multiple hospital presentations required for this type of therapy.^19^^,^^44^^,^^50^ Brachytherapy, on the other hand, typically requires only a single-day procedure for ^32^P microparticle implantation and is therefore less burdensome. This represents a substantial advantage in patients with mPDAC, in whom maximizing QoL and minimizing hospital admissions are valued.

We observed a good safety profile for ^32^P microparticle implantation in this cohort, with no grade 4/5 toxicities, and no grade 3 toxicities attributable to the implantation procedure or local radiation effects. This is despite delivering 100 Gy absorbed dose into the target tumor, while sparing surrounding healthy tissue.^51^^,^^52^ As discussed, this dose is much higher than with conventional radiotherapy or SBRT (25-60 Gy), and the likely reason for its tolerability is that ^32^P emits beta rays that travel only a short distance within tissue (<1 cm) and are therefore much less likely to affect surrounding healthy tissues. Moreover, with its half-life of 14.3 days, ^32^P implanted microparticles emit radiation continuously over approximately 72 days rather than being delivered in short periods over a number of discrete fractions. Data suggest that a high dose of radiotherapy is required to achieve adequate tumor control, and thus the ability to deliver such a high dose without compromising its safety profile is a major advantage.^53^^,^^54^

We acknowledge that the current study has limitations, including a retrospective design, small sample size, and lack of a comparative arm. With the retrospective design, and ^32^P microparticle implantation being novel therapy given to a small number of patients, there is likely treatment selection bias. As a result of being a single-arm trial, only the effects of combination chemotherapy can be presented rather than the benefit of ^32^P implantation plus chemotherapy over chemotherapy. However, it has shown positive outcomes that would support the undertaking of a larger comparative prospective study.

In conclusion, this represents the first report of the effect of combining standard-of-care chemotherapy with EUS-guided ^32^P microparticle implantation in patients with mPDAC. We observed encouraging outcomes, including 100% LDCR at 3 months’ postimplantation. This retrospective analysis highlights the potential clinical benefits, particularly for local tumor control while avoiding compromising QoL, in a patient cohort with historically poor outcomes.

## Disclosure

The following authors disclosed financial relationships: H. Wasan: advisory boards/invited speaker/meetings for Servier, Pierre Fabre, Incyte, Bayer, Pfizer, Zymeworks, Merck KGaA, Seagen, Exact Therapeutics, Takeda (HUTCHMED), Amgen/Roche/Genentech/FM, Sirtex Medical, Erytech Pharma, BMS (Celgene), and BTG; consultancies/advisory role for NICE/BSI, Bayer, Pierre Fabre, OncoSil Medical Limited, Incyte, Celgene, and Oaktree Life Sciences; global trials committees for Pfizer (Array), Zymeworks, Boehringer Ingelheim (DMC), Sirtex, Merck KGaA, and ARCAD (Pancreas Academic); and research funding from 10.13039/100030732MSD, Merck Serono, 10.13039/100004319Pfizer, and Sirtex. D. Croagh: shareholder in Margin Clear. All other authors disclosed no financial relationships.
